# Relationship between Platelet PPARs, cAMP Levels, and P-Selectin Expression: Antiplatelet Activity of Natural Products 

**DOI:** 10.1155/2013/861786

**Published:** 2013-11-13

**Authors:** Eduardo Fuentes, Iván Palomo

**Affiliations:** ^1^Department of Clinical Biochemistry and Immunohematology, Faculty of Health Sciences, Programa de Investigacion de Excelencia Interdisciplinaria en Envejecimiento Saludable (PIEI-ES), Universidad de Talca, 3460000 Talca, Chile; ^2^Centro de Estudios en Alimentos Procesados (CEAP), CONICYT-Regional, Gore Maule, R09I2001 Talca, Chile

## Abstract

Platelets are no longer considered simply as cells participating in thrombosis. In atherosclerosis, platelets are regulators of multiple processes, with the recruitment of inflammatory cells towards the lesion sites, inflammatory mediators release, and regulation of endothelial function. The antiplatelet therapy has been used for a long time in an effort to prevent and treat cardiovascular diseases. However, limited efficacy in some patients, drug resistance, and side effects are limitations of current antiplatelet therapy. In this context, a large number of natural products (polyphenols, terpenoids, alkaloids, and fatty acids) have been reported with antiplatelet activity. In this sense, the present paper describes mechanisms of antiplatelet action of natural products on platelet P-selectin expression through cAMP levels and its role as peroxisome proliferator-activated receptors agonists.

## 1. Introduction

Cardiovascular diseases (CVD) result in >19 million deaths annually and coronary heart disease accounts for the majority of this toll. Actually a large number of victims of the disease who are apparently healthy die suddenly without prior symptoms [1]. The incidence and prevalence of CVD have increased significantly in recent years [2–4] and are regulated by both genetic and environmental factors (dyslipidemia, hypertension, smoking, diabetes, and obesity) [5, 6].

Platelet accumulation at sites of vascular injury is the primary event in arterial thrombosis and the activation is a critical component of atherothrombosis [7]. Thus patients with unstable complex lesions had a fivefold higher expression of the platelet activation epitope CD63 than patients with stable angina, indicating an intense thrombogenic potential [8]. Platelets also interact directly with other cells of the immune system in physiological and pathological conditions [9, 10]. Platelet-derived P-selectin seems to contribute to atherosclerotic lesion development and arterial thrombogenesis by forming large stable platelet-leukocyte aggregates [11]. In this context, the percentage of neutrophil-platelet conjugates increased by 22% in patients with unstable angina pectoris [12]. Also platelets can be directly involved in the plaque unstable by the production and release of proinflammatory molecules, including a variety of cytokines, such as TGF-*β*, IL-1*β*, and sCD40L, and chemokines, such as CXCL7, CXCL4, CXCL4L1, CCl5, CXCL1, CXCL8, CXCL5, CXCL12, CCL2, and CCL3 [13, 14].

The antiplatelet therapy has been used for a long time in an effort to prevent and treat CVD [15, 16]. However, limited efficacy in some patients, drug resistance, and side effects are limitations of current antiplatelet therapy [17, 18]. Therefore, there is much room for further improvement of antiplatelet treatment and search of novel antiplatelet agents with increased efficacy and safety profile. In this context, a large number of natural products (polyphenols, terpenoids, alkaloids, and fatty acids, among others) have been reported with an inhibitory activity on platelets function [19].

Interestingly, some natural compounds consumed regularly in the diet may have protective effects in primary and secondary prevention of CVD [20, 21]. In this context, a great deal of interest has been paid by consumers towards natural bioactive compounds as functional ingredients in diets due to their various beneficial health effects [22–25]. Natural bioactive compounds from fruit, vegetables, beverages, and grass among others have antiplatelet effects and may thus affect the development of CVD [26].

In this sense, the present paper describes mechanisms of antiplatelet action of natural products by PPARs signaling pathway and inhibit of platelet P-selectin expression through of cAMP.

## 2. Regulation of Platelet cAMP Levels by PPARs

The PPARs consist of three nuclear receptor isoforms (*γ*, *β*/*δ*, and *α*) [27]. PPARs are key regulators of metabolic syndrome and play an important role in the processes that govern chronic inflammatory diseases [28, 29]. Thus PPARs remain attractive therapeutic targets for the development of drugs used in the treatment of chronic inflammatory diseases such as atherosclerosis [30]. PPAR-*δ* antagonizes multiple proinflammatory pathways [31] and is pivotal to control the program for fatty acid oxidation in the skeletal muscle [32]. 

PPARs modulate atherosclerosis development by acting at both metabolic and vascular levels [33]. Thus PPARs activation is a key mechanism for improving cardiovascular function resulting from weight loss [34–36]. PPARs are expressed in human platelets [37]. In this context, PPARs appear to play a major role in the regulation of atherogenesis by countering the inflammation-provoking action of platelet adhesion and activation [38]. The antiplatelet activity of statins and fibrates on platelet function is mediated by PPARs activation via a novel mechanism that involves the inhibition of protein kinase-*α* (PKC-*α*) [39]. In addition, statins by increasing both cAMP as well as cGMP pathways could inhibit platelet activation [39]. cAMP increased by PPAR activation is due to the repression of PKC that allows greater activity of adenylyl cyclase (ATP to cAMP) [40, 41]. Meanwhile, cAMP-induced inhibition of platelet P-selectin expression is through activation of protein kinase A (PKA) [42]. 

## 3. Relationship between cAMP Levels and Platelet P-Selectin Expression

It has been shown that cAMP and cGMP-dependent protein kinases not only inhibit platelet pathways leading to activation and aggregation, but also those resulting in enhanced surface expression of protein ligands involved in inflammation [43]. Also, Ca^2+^ in human platelets is directly downregulated by cGMP and cAMP by a mechanism involving the inhibition of cytoskeletal reorganization via the activation of protein tyrosine phosphatases [44]. 

Moreover, platelet shape change can be antagonized by PKA (cAMP-dependent) activation but not by protein kinase G (PKG) (cGMP-dependent), which may occur with particular efficiency by the formation of a local compartment of cAMP through the inhibition of phosphodiesterase-3 (PDE3) [45]. In fact, activation/phosphorylation of PDE3 via Akt signaling pathway participates in regulating cAMP during thrombin activation of platelets [46]. Together, these results indicate that cAMP is persistently formed in platelets [47].

cAMP-induced inhibition of platelet P-selectin expression is, in large part, mediated through the activation of PKA [42]. While P-selectin expression was found to be independent of mitogen-activated protein kinase (MAPK) activation, since it was not inhibited by specific MAPK inhibitors [43]. Inhibition of ADP-induced P-selectin expression and platelet-leukocyte conjugate formation was inhibited by clopidogrel and AR-C69931MX but not by aspirin [48, 49]. Prolonged cyclooxygenase-2 (COX-2) inhibition attenuates C-reactive protein and IL-6, but does not modify P-selectin [50]. ARC69931MX and clopidogrel by cAMP levels can inhibit human platelet aggregation through the activation of a separate G protein-coupled pathway (presumably involving Gs) and platelet P2Y12 receptor, respectively [51, 52]. Andersen et al. showed that levels of soluble P-selectin were significantly higher in aspirin responders and nonresponders [53]. Despite the above, measurement of circulating P-selectin has been suggested for remote testing of platelet function in patients treated with clopidogrel and aspirin [54]. 

## 4. Mechanism of Antiplatelet Action of Natural Products 

In the context of atherosclerosis CVD, platelets can adhere to endothelial cells and leukocytes and contribute to vascular inflammation and thrombosis formation [55, 56]. In this sense, the inhibition of the platelet function has been used for long time in an effort to prevent and treat CVD [57]. However, limited efficacy in some patients, drug resistance, and side effects are limitations of current antiplatelet therapy [17, 18]. Moreover, epidemiological studies have provided evidence of a protective role of healthy diets in the prevention of CVD [58, 59]. 

The consumption of a diet containing 30% green and yellow vegetables results in a substantial inhibition of atherosclerosis progression [60]. Preliminary studies have demonstrated the platelet antiaggregation activity of fruit (red grapes, strawberries, kiwis, and pineapples) and vegetables (garlic, onions, green onions, melons, and tomatoes) [61, 62]. In this context, consuming two or three kiwi fruits per day for 28 days reduces platelet aggregation induced by collagen and ADP [63]. Strawberries are likely to exert significant protective effects in thromboembolic-related disorders by inhibiting platelet aggregation [64, 65]. Organo sulfur compounds in onion extracts are formed following the lysis of the S-alk(en)yl-L-cysteine sulfoxides by alliinase. These compounds inhibit the aggregation of human blood platelets and offer the potential for positive cardiovascular health benefits [66]. The raw form of garlic and some of its preparations are widely recognized as antiplatelet agents that may contribute to the prevention of CVD. Antithrombotic activities of garlic have been demonstrated by blood fibrinolytic and coagulation systems, and inhibition of platelet aggregation [67]. With respect to platelet function, allicin and thiosulfinates are responsible for *in vitro* antiaggregatory activity from garlic [68]. Furthermore, recently galactolipid and a phytosterol from garlic were identified as exhibiting an inhibitory action on ADP-induced aggregation in human blood platelets [69].

In fact, a large number of natural products have been reported with apparent inhibitory activity on human platelets and each constituent may possess multiply targets, and they may exert pleiotropic and synergistic effects (Table 1) [70–72].

### 4.1. Antiplatelet Activity of Natural Products by PPARs

Due to high levels of toxicity associated with the first generation of drugs, there is renewed search for newer PPAR drugs that exhibit better efficacy but lesser toxicity [110]. Moreover, there has been a definite increase in the consumption of fruits and vegetables, due to the possible health benefits associated with these bioactive components [74, 111]. Thus, dietary components that act as ligands of PPARs include dietary lipids such as n-3 and n-6 fatty acids and their derivatives, polyphenols, and terpenoids, among others [112–114] (Table 2).

**Figure 1:**
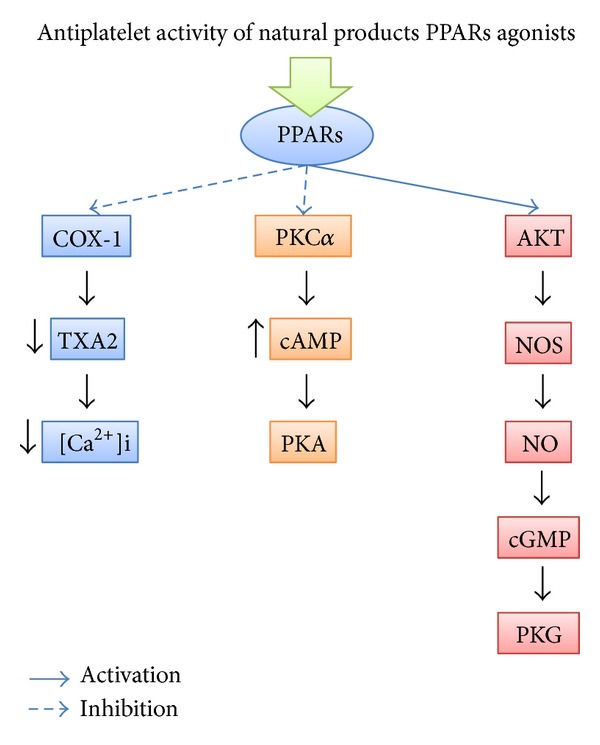
Mechanism of antiplatelet action by natural products on PPARs. cAMP = cyclic adenosine monophosphate; PKA = protein kinase A; TXA2 = thromboxane A2; PKC = protein kinase C; PLC = phospholipase; COX-1 = cyclooxygenase-1; PPARs = peroxisome proliferator-activated receptors; AKT = also known as protein kinase B; NO = nitric oxide; cGMP = cyclic guanosine monophosphate; PKG = protein kinase G; NOS = nitric oxide synthase.

In this sense, the present paper describes the mechanism of antiplatelet action of natural products as PPARs agonists and increased of intraplatelet levels of cAMP. As shown in Figure 1, the mechanism of antiplatelet action by natural products PPARs agonists is mediated by the following signaling pathways: (i) inhibition of PCK-*α*/increased of cAMP levels/stimulation of PKA (increased of cAMP levels), (ii) stimulation of Akt/NOS/NO/PKG (increased of cGMP levels), and (iii) inhibition of cyclooxygenase-1 (COX-1), thromboxane A2 (TXA2), and Ca^2+^ mobilization.

Magnolol is the major bioactive constituent of *Magnolia officinalis* (2–11% of the bark's dry weight) [115, 116]. Magnolol could improve insulin sensitivity through the activation of PPAR-*γ* [117]. Also Magnolol presents antiplatelet activity by PPAR-*β*/*γ* activation with upregulation of Akt/NOS/NO/cGMP/PKG cascade and suppression of PKC-*α* and COX-1 and Ca^2+^ mobilization [96]. 

Linolenic acid impairs arterial thrombus formation, tissue factor expression, and platelet activation and thereby represents an attractive nutritional intervention with direct dual antithrombotic effects [118]. These effects could be because both oleic and linoleic acids are PPARs agonists [119]. Meanwhile *α*-lipoic acid is PPAR-*α*/*γ* agonist and the mechanism of action involves the inhibition of Ca^2+^ mobilization, TXA2, PKC-*α*, and COX-1 expression, and elevation of cAMP levels [104, 105].

*α*- and *γ*-tocopherols have been shown to activate PPAR-*γ* expression and *γ*-tocopherol is a better modulator of PPAR-*γ* expression than *α*-tocopherol [106, 107]. In this context, *α*-tocopherol inhibits platelet aggregation through a PKC-dependent mechanism, which may explain a decrease in the expression of P-selectin and interactions platelet-mononuclear cells *ex vivo* [108, 109]. 

Curcumin, the major component of food spice turmeric (*Curcuma longa*), inhibits platelet aggregation induced by PAF and arachidonic acid with inhibitory effects on TXA2 and Ca^2+^ mobilization and also prevents the adhesion of platelets to brain microvascular endothelial cells [84–86]. The beneficial effect of curcumin on platelet activation appears to be mediated by the upregulation of PPAR-*γ* [87].

### 4.2. Antiplatelet Activity of Natural Products by cAMP Levels

Here we describe one possible mechanism of action of natural products on platelet P-selectin expression through cAMP.

The natural products caffedymine (clovamide-type phenylpropenoic acid amide found in cocoa), N-caffeoyl tyramine, N-feruloyl tyramine, 5-caffeoylquinic acid, caffeic acid, and gallic acid were able to suppress P-selectin expression on platelets and were found to be very potent compounds able to inhibit COX-1 and 2 enzymes [73, 78, 81, 120–122]. Moreover, previous studies indicate that caffedymine and N-caffeoyl tyramine inhibit P-selectin expression by increasing cAMP through beta-2 adrenoceptors [79, 80, 123]. Gallic acid, in a concentration-dependent manner, prevents the elevation of intracellular calcium and attenuate phosphorylation of PKC*α*/p38 MAPK and Akt/GSK3*β* on platelets stimulated by the stimulants ADP or U46619 [70]. Based on the function of other cell (mast cells), the mode of action of gallic acid is likely related with the elevation of the intracellular cAMP level by the inhibition of the cAMP phosphodiesterase [124].

Adenosine is another natural product with antiplatelet activity [74, 75]. Adenosine through G-protein linked receptors to activate adenylate cyclase and increase cellular cAMP levels, showing the inhibition of platelet P-selectin expression [76, 77]. However, chlorogenic acid, an antiplatelet compound, presented increase of cAMP and cGMP levels and strong inhibition of COX-1 [125] and COX-2 [126] but did not have effect on P-selectin expression [127].

Moreover, sanguinarine, alkaloid present in the root of *Sanguinaria canadensis* and *Poppy fumaria* species, is a potent antiplatelet agent, which activates adenylate cyclase with cAMP production, inhibits platelet Ca^2+^ mobilization and TXA2 production as well as suppresses COX-1 enzyme activity (whereas its effect on COX-2 activity was minimal) [99]. Similar antiplatelet effect had girinimbine that presented the inhibition of COX activity and elevation of the cAMP level [93]. 

Being increased of intraplatelet levels of Ca^2+^ involves phosphorylation of both pleckstrin (47 kDa) and myosin light chain (20 kDa) via Ca^2+^-dependent PKC and Ca^2+^/calmodulin-dependent protein kinase (CaM-PK), respectively. The phosphorylation of these proteins participates in the release of platelet aggregation factors such as serotonin and ADP [128, 129]. In this context, the effect of cordycepin on platelet aggregation might be associated with the inhibition of phosphorylation of these proteins to suppress the release of serotonin and ADP out of dense body in platelets, which is associated with the inhibition of Ca^2+^ mobilization by cordycepin-elevated cAMP [82, 83]. Whereas the ODQ (NO-sensitive guanylyl cyclase inhibitor) did not alter the cordycepin-induced upregulation of cGMP, the adenylyl cyclase inhibitor SQ22536 completely blocked the cAMP enhancement mediated by cordycepin [82]. Sulforaphane possesses potent antiplatelet activity, which may initially activate adenylate cyclase/cAMP, followed by inhibiting intracellular signals (such as the PI3-kinase/Akt and PLC*γ*2-PKC-p47 cascades) [102, 103]. Furthermore epigallocatechin-3-gallate increases cAMP via adenylate cyclase activation and subsequently phosphorylates VASP-Ser-157 through A-kinase activation to inhibit Ca^2+^ mobilization and TXA2 production on collagen-induced platelet aggregation [91]. Sesamol possesses potent antiplatelet activity, which may involve the activation of the cAMP-eNOS/NO-cGMP pathway, resulting in the inhibition of the PLC*γ*2-PKC-p38MAPK-TXA2 cascade [100]. Also, sesamol activates cAMP-PKA signaling, followed by the inhibition of the NF-*κ*B-PLC-PKC cascade. The inhibition of NF-*κ*B which interferes with platelet function may have a great impact when these types of drugs are considered for the treatment of cancer and various inflammatory diseases [101]. The inhibition of platelet aggregation by *α*-lipoic acid is mediated by PPAR*α*/*γ*-dependent processes, which involve interaction with PKC and COX-1, increase of cAMP formation, and inhibition of intracellular Ca^2+^ mobilization [104]. However, the effects of *α*-lipoic acid on the above platelet responses were markedly reversed by the addition of 2′5′-ddAdo, an adenylate cyclase inhibitor [105]. Meanwhile, quercetin-mediated antiplatelet activity involves PI3K/Akt inactivation, cAMP elevation, and VASP stimulation that, in turn, suppresses MAPK phosphorylations [98]. Intraplatelet cAMP production was quickly increased by quercetin stimulation and probably through the adenylate cyclase signaling pathway [130].

According to natural products as caffedymine, N-caffeoyl tyramine, quercetin, and adenosine, which increase the intraplatelet cAMP levels and inhibit platelet P-selectin expression. It is possible to consider that those natural products (sanguinarine, *α*-Lipoic acid, sesamol, sulforaphane, epigallocatechin-3-gallate, and cordycepin) which increase the intraplatelet cAMP levels and lose their antiplatelet activity after adenylate cyclase blockaded would be able to inhibit platelet P-selectin expression. Even only an increase in the intraplatelet cAMP Levels may establish that dicentrine and girinimbine could inhibit P-selectin expression. Thus, the relationship between cAMP levels and P-selectin expression is because cAMP via the activation of PKA is capable of inhibiting platelet P-selectin expression [42, 77]. Furthermore, natural products that inhibited platelet aggregation stimulated by ADP and collagen with increased of cAMP levels is because cAMP downregulates P2Y1R expression [131] and GPVI-maintained in a monomeric form on resting platelets [132].

**Figure 2:**
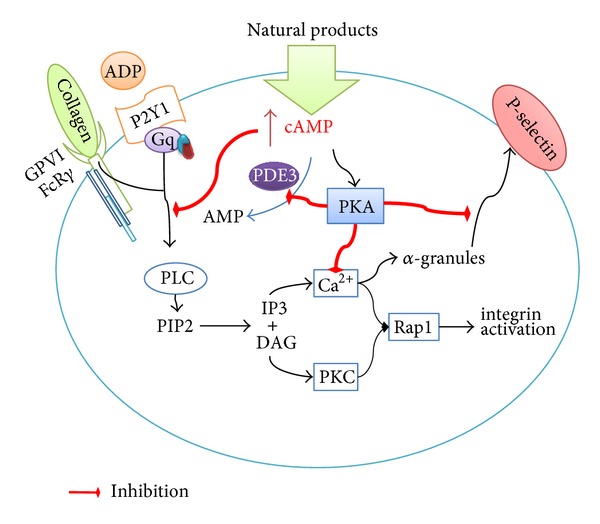
Mechanism of antiplatelet action of natural products by cAMP levels. PDE3: phosphodiesterase-3; PKA: protein kinase A; PLC: phospholipase; DAG: diacylglycerol; IP3: inositol trisphosphate; PIP2: phosphatidylinositol 4,5-bisphosphate; PKC: protein kinase C; PSGL-1: P-selectin glycoprotein ligand-1.

Finally, it is possible to establish that natural products that show antiplatelet activity by increasing levels of cAMP are able to inhibit platelet-leukocyte interactions through P-selectin inhibition (Figure 2). This makes it possible to consider that natural products in addition to platelet function inhibitors are compounds capable of preventing atherothrombosis/atheroinflammation.

## 5. Conclusions

According to this paper it is possible to establish that the antiplatelet activity by PPARs agonist and increased cAMP levels are not defined by one specific group of bioactive compounds. Also the data presented in this paper demonstrate that natural products with antiplatelet activity through of increase cAMP levels are able to inhibit the platelet-leukocyte interactions in atheroinflammation.

## Figures and Tables

**Table 1:** Antiplatelet effects induced by various agonists of natural products and mechanisms described.

Constituents	Platelet aggregation									Mechanisms	References
	AA	Collagen	U46619	Thrombin	PAF	ADP	Epinephrine	A23187	TRAP-6		
5-Caffeoylquinic acid	/	/	/	/	/	/	/	/	/	Inhibition of COX-1, COX-2, and P-selectin expression.	[73]
Adenosine				+		+				cAMP and cGMP production. Inhibition of P-selectin expression.	[74–77]
Caffedymine (N-caffeoyldopamine)	/	/	/	/	/	/	/	/	/	Inhibition of TXA2, COX-1, COX-2, and P-selectin expression. cAMP production.	[78–80]
Caffeic acid	/	/	/	/	/	/	/	/	/	Inhibition of COX-1, COX-2, and P-selectin expression.	[73, 81]
Cordycepin	/	+	+	/	/	/	/	/	/	Inhibition of [Ca^2+^]i and TXA2. cAMP and cGMP production.	[82, 83]
Curcumin	+	/	/	/	+	/	/	/	/	PPAR-*γ* activation, inhibition of [Ca^2+^]i and TXA2.	[84–87]
C-phycocyanin	−	+	+	+	/	/	/	/	/	Inhibition of TXA2, PDE3, PKC, [Ca^2+^]i. cGMP production.	[88, 89]
Dicentrine	+	+	+	+	+	+	/	/	/	Inhibition of [Ca^2+^]i and TXA2. cAMP production.	[90]
Epigallocatechin-3-gallate	/	+	/	/	/	−	−	/	/	Inhibition of [Ca^2+^]i, TXA2. cAMP production.	[91]
Flavonoid alpha-naphthoflavone	+	+	/	−	/	+	/	/	/	Inhibition of [Ca^2+^]i, TXA2, PLC, PKC and phosphoinositide breakdown. cGMP production.	[92]
Gallic acid	/	/	+	/	/	+	/	/	/	Inhibition of [Ca^2+^]i, P-selectin expression, PKC*α*/p38 MAPK and Akt/GSK3*β*.	[70]
Girinimbine	+	+	+	−	+	/	/	/	/	Inhibition of TXA2, PGD2, PGE2, and [Ca^2+^]i. cAMP production.	[93]
Hesperetin	+	+	−	−	/	/	/	/	/	Inhibition of PLC-*γ*2, [Ca^2+^]i and COX-1.	[94]
Hydroxychavicol	+	+	/	−	/	/	/	/	/	Inhibition of [Ca^2+^]i, TXA2, ROS production, COX-1, and COX-2.	[95]
Magnolol	+	+	/	/	/	/	/	/	/	PPAR-*β*/*γ* activation, upregulation of Akt/NOS/NO/cGMP/PKG and inhibition of PKC-*α*, COX-1, and Ca^2+^ mobilization	[96]
N-caffeoyl tyramine (N-coumaroyldopamine)	/	/	/	/	/	/	/	/	/	Inhibition of TXA2, COX-1, COX-2, and P-selectin expression. cAMP production.	[78–80]
Phloroglucinol	+	/	−	/	/	/	/	/	/	Inhibition of TXA2, ERK/p38, ROS production COX-1, and COX-2 activities.	[97]
Quercetin	/	+	/	+	/	+	/	/	/	Inhibition of [Ca^2+^]i, P-selectin expression, GPIIb/IIIa, PI3K, Akt, ERK2, JNK1, and p38 MAPK. cAMP and VASP production.	[98]
Sanguinarine	+	+	+	+	/	/	/	/	/	Inhibition of [Ca^2+^]i, TXA2, COX-1, and COX-2. cAMP production.	[99]
Sesamol	+	+	−	−	/	/	/	/	/	Inhibition of [Ca^2+^]i, TXA2, PLC-*γ*2, PKC, MAPK, and NF-*κ*B signaling events. cAMP, cGMP, and NO production.	[100, 101]
Sulforaphane	−	+	+	−	/	+	/	/	/	Inhibition of [Ca^2+^]i, PLC-*γ*2, PKC, MAPKs, and PI3K/Akt. cAMP production.	[102, 103]
*α*-Lipoic acid	+	+	/	/	/	/	/	/	/	PPAR-*α*/*γ* activation, inhibition of [Ca^2+^]i, TXA2, PKC*α*, and COX-1. cAMP production.	[104, 105]
*α*- and *γ*-Tocopherol	+	/	/	/	/	+	/	/	/	PPAR-*γ* activation, inhibition of PKC, and P-selectin expression.	[106–109]

“+”: positive antiplatelet effect; “−”: no or little antiplatelet effect; “/”: not reported; AA: arachidonic acid; PAF: platelet-activating factor; COX: cyclooxygenase; PLC: phospholipase C; PKC: protein kinase C; ROS: reactive oxygen species; NO: nitric oxide; TXA2: thromboxane A2; PGD2: prostaglandin D2; MAPKs: mitogen-activated protein kinase.

**Table 2:** Natural products PPARs agonists.

PPAR-*α*	
Catalposide	
Berberine	
Astaxanthin	
9-Oxo-octadecadienoic acid	
PPAR-*γ*	
Artepillin C	
Kaempferol	
20S-protopanaxatriol	
Apigenin	
Quercetin	
6-Shogaol	
Chrysin	
(−)-catechin	
Harmine	
3-Acetyl oleanolic acid	
9S,13R-12-oxo-phytodienoic acid	
Auraptene	
Oleic acid	
PPAR-*α*/*γ*	
Cyanidin	
Vaccenic acid	
